# Specificity of mRNA binding to proteins within the NMD machinery is influenced in cancer

**DOI:** 10.3389/fmolb.2025.1606190

**Published:** 2025-10-20

**Authors:** Umesh Kalathiya, Monikaben Padariya

**Affiliations:** International Centre for Cancer Vaccine Science, University of Gdansk, Gdansk, Poland

**Keywords:** premature stop codon, cancer, genetic diseases, mRNA, mutation, NMD

## Abstract

**Introduction:**

The nonsense-mediated mRNA decay (NMD) process is recognized as the quality control of mRNAs to maintain their integrity and production of functional proteins. Readthrough of aberrant mRNA containing premature termination codons (PTCs) can induce the production of truncated proteins with negative functionalities.

**Methods:**

To elucidate the structural and mechanistic basis of NMD components, we performed molecular dynamic simulations (MDS) to analyze their dynamic behavior across different stages of the process. We further investigated how cancer-associated mutations alter mRNA-binding protein (RBP) interactions within the NMD machinery.

**Results and Discussion:**

Over the simulation time, the mRNA containing PTCs underwent significant conformational rearrangements, ultimately forming stable interactions with the eukaryotic class-I release factor (eRF1). The efficiency of eRF1 in recognizing stop codons (UAG, UGA, or UAA) nitrogenous bases was identified, revealing a stronger preference toward UAA. Due to the lower structural stability, the AU-rich mRNA motifs showed a diminished eRF1 binding affinity relative to other PTC-containing transcripts. Among the studied cancer variants, the D9Y, R10S, F56V, P89L, and I62M residues were found to either enhance or disrupt eRF1–mRNA interactions. Similarly, when evaluating EIF4A3 RBP from the exon junction complex (EJC), the P114L and G309A mutations significantly impaired the protein–mRNA binding affinity. Surface residue mapping of SMG1 kinase revealed that it engages with SMG8, SMG9, and UPF1 in a sequential binding order, displaying the highest affinity for SMG8. Overall, these findings contribute to the mechanistic understanding of molecular properties for different RBPs from the NMD process, which can be the basis of developing new therapeutic strategies against genetic disease or cancer.

## 1 Introduction

The quality or integrity of mRNA that results in the production of functional protein is controlled by different mechanisms, and one among them is the nonsense-mediated mRNA decay process (NMD) (Tyler, 2016). When a premature termination codon (PTC) is detected in mRNA, ribosome translation stalls, leading to the activation of the NMD pathway (Melero et al., 2014; Padariya and Kalathiya, 2022; Hug et al., 2016). This machinery has been implicated in several crucial cellular processes (Melero et al., 2014), including cell cycle regulation, gene expression control, DNA damage response (DDR), and tissue homeostasis. PTCs in mRNA could occur due to mutations from a single nucleotide that convert a canonical triplet (UAA, UGA, and UAA) nucleotide codon. Whereas NMD degrades PTC-containing mRNAs, any transcripts that evade degradation can produce potentially harmful truncated proteins with dominant-negative activity (Maquat et al., 1981). The NMD process has a crucial role in targeting ∼10% of unmutated mammalian mRNAs, thus acting as a regulator of cellular adaptation to environmental changes and cell survival (Hug et al., 2016). Among the other components involved in the NMD process, the RNA-dependent ATPase UPF1 (up-frameshift protein 1) serves as the master regulator of the machinery.

**FIGURE 1:**
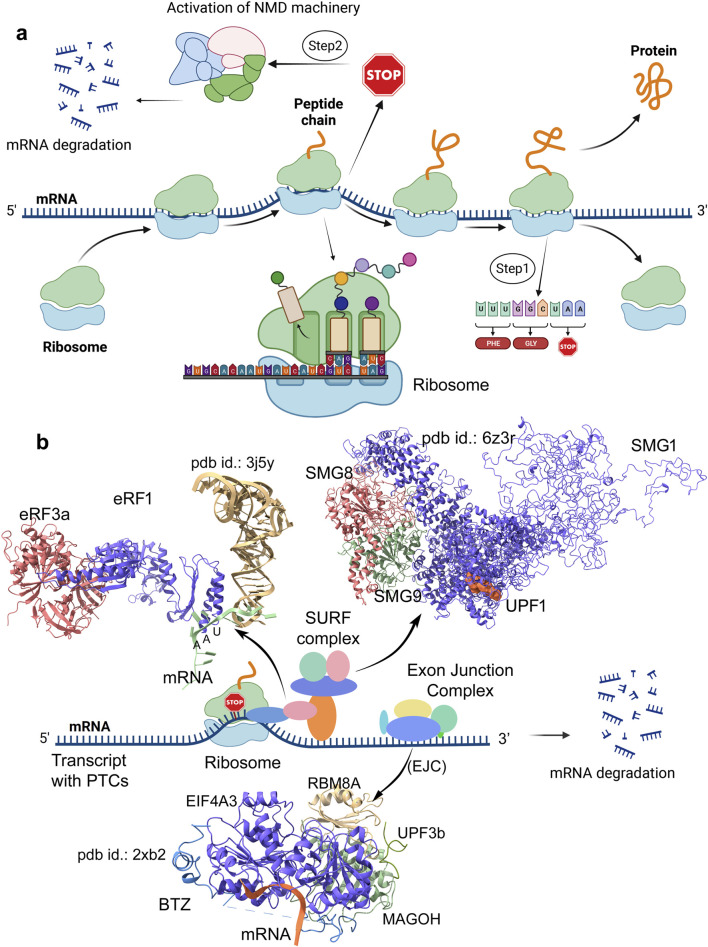
Schematic representation of mRNA transcripts processed in the presence or absence of premature termination codons (PTCs), along with demonstrating protein complexes from NMD (nonsense-mediated mRNA decay process) involved in docking mRNA at different sites. **(a)** Outline of translation of mRNA lacking PTCs to protein production is defined as step 1. In addition, step 2 illustrates the involvement of the stalled ribosome in the process upon the detection of mRNA containing PTCs, resulting in the activation of the NMD machinery, which eventually causes mRNA degradation. **(b)** Modeled structures by inserting missing residues for SURF components (SMG1–SMG8–SMG9-UPF1; pdb id.: 6z3r (Langer et al., 2020)), exon junction complex (EJC; Mago–Y14–eIF4AIII–Barentsz–UPF3b assembly; pdb id.: 2xb2 (Buchwald et al., 2010)), and the eRF complex (eRF1–eRF3a; pdb id.: 3j5y (des Georges et al., 2014)). These diagrams were prepared and designed using the BioRender platform (www.biorender.com).

The potential players of the NMD pathway involve the formation of different sub-complexes, including the exon junction complex (EJC) composed of EIF4A3 (eukaryotic initiation factor 4A3), MAGOH (Mago nashi homolog), Y14 or RBM8A (RNA-binding motif protein 8A), and Barentsz (Figure 1). The SURF complex consists of UPF1, SMG1 (serine/threonine-protein kinase), eRF1 (eukaryotic translation termination factor 1), and eRF3. The decay-inducing complex (DECID) is formed by UPF1-3b, SMG1, SMG8, and SMG9 (Hug et al., 2016). The recruitment of UPF proteins to the EJC is a crucial step in activating the NMD process (Gardner, 2010). During the initial rounds of mRNA translation, a few genes are relocated by the ribosome, and such conformational changes by EJC are stored until the mRNA is translated (Dostie and Dreyfuss, 2002; Dreyfuss et al., 2002). Approximately at 20–24 nucleotides (nt) upstream of the spliced exon–exon junction, the EJC on the mRNA is assembled, and the protein–mRNA interactions occur in a splicing dependent type (Le Hir et al., 2000). Consisting of two RecA-like domains, the *EIF4A3* gene interacts with mRNA within the EJC complex. Notably, in *EIF4A3*, the ATP binding creates a contiguous cleft that facilitates RNA binding (Tyler, 2016). The interaction of Barentsz (BTZ) with EIF4A3, MAGOH, and RBM8A proteins from the EJC stabilizes the mRNA binding (Ballut et al., 2005).

The detection of PTC-containing transcripts that cause stalled translation (upstream of EJC) enhances the binding of eukaryotic release factors to the mRNA, recruiting the UPF1 helicase (Lykke-Andersen et al., 2000; Lejeune et al., 2003). Later, the SMG1 phosphorylates UPF1, forming the SURF complex (Kashima et al., 2006), due to which the master regulator of the NMD machinery recruits SMG5, SMG6, and SMG7 (Fukuhara et al., 2005). In addition, the phosphorylated residues in the C-terminus of UPF1 are found to interact with SMG5 and SMG7 (Okada-Katsuhata et al., 2012; Jonas et al., 2013). In mammalian cells, SMG6 is widely regarded as the principal endonuclease that cleaves PTC-containing mRNAs (Huntzinger et al., 2008). The SURF components dock with UPF2, UPF3b, and an EJC downstream of the PTC, which is involved in the DECID complex. Alterations in SMG1 impair this phosphorylation event and the interactions with SMG8, SMG9, eRF1, and eRF3, resulting in defective NMD and the inability to identify mRNAs containing PTCs (Kashima et al., 2006; Eberle et al., 2009; Dana and Tuller, 2014; Bongiorno et al., 2021). Furthermore, the eRF1–eRF3a structure was resolved with the ribosomal pre-termination complex (Popp and Maquat, 2016; des Georges et al., 2014). In addition, several protein complex structures associated with the NMD machinery have been structurally classified and are available in the protein data bank (pdb) (Rose et al., 2011), including UPF1–UPF2 (pdb id.: 2wjv) (Clerici et al., 2009), UPF2–UPF3 (pdb id.: 1uw4) (Kadlec et al., 2004), SMG5–SMG7 (pdb id.: 3zhe) (Jonas et al., 2013), SMG8–SMG9 (pdb id.: 5nkk) (Li et al., 2017), SMG1–SMG8–SMG9–UPF1 (pdb id.: 6z3r and 6syt) (Gat et al., 2019; Langer et al., 2020), Mago–Y14–EIF4A3–Barentsz–UPF3b (pdb id.: 2xb2) (Buchwald et al., 2010), and eRF1–eRF3a (pdb id.: 3j5y) (des Georges et al., 2014).

Within the NMD machinery, key proteins including UPF1 (Muhlrad and Parker, 1994), EIF4A3 (Buchwald et al., 2010), and eRF1 (des Georges et al., 2014) directly mediate mRNA docking. Unlike the bacterial class-I release factors where RF1 recognizes UAG/UAA and RF2 recognizes UGA/UAA, the human eRF1 (ETF1) recognizes all three stop codons (UAG, UGA, and UAA) (Kisselev et al., 2003; Cheng et al., 2009). eRF1 is a critical player in protein synthesis, making it a preferred therapeutic target for modulation *via* small molecules, aptamers, or peptidomimetics. The overexpression of EIF4A3 in different cancers has the potential to drive tumor progression by regulating RNA splicing, translation, and nonsense-mediated decay. Earlier studies have demonstrated that its knockdown results in the activation of tumor-suppressor genes (Blázquez-Encinas et al., 2024; Miliara et al., 2024), making it a potential target for RNA-based therapies or inhibitors. Additionally, studies have resolved the interactions of UPF1 and EIF4A3 with poly(U) mRNA, providing further mechanistic insights into NMD regulation (Buchwald et al., 2010; Muhlrad and Parker, 1994). UPF1’s binding specificity for GC- and AU-rich mRNAs has been well characterized, with studies demonstrating its direct influence on catalytic activity and versatility in mRNA recognition (Padariya et al., 2021; Li et al., 2014; Perry and Ulitsky, 2016; Brown et al., 2014). SMG1 is a key kinase in NMD that is found to interact with SMG8 and SMG9, which regulate its activity and UPF1 phosphorylation (Figure 1) (Langer et al., 2020; Kurosaki et al., 2016; Zhu et al., 2019). Dysregulated SMG1–UPF1 activity can be targeted with small molecules for NMD-modulating cancer therapies (Leeksma et al., 2023).

Comprehending the NMD process is dependent on a few specific protein–protein and protein–mRNA interactions that are governed by structural and physicochemical properties. Even minor perturbations such as amino acid substitutions, insertions, or deletions can disrupt binding interactions, alter affinity and specificity, or shift binding modes (Guerois et al., 2002; Kamburov et al., 2015). These effects are particularly consequential in cancer and genetic disorders, characterized by premature translation termination or dysregulated protein synthesis. Given the crucial role of mRNA-binding complexes in NMD for proper function and mRNA containing PTC detection, we investigated their structural dynamics at the molecular level and their implications in cancer mechanisms. Our findings contributed in defining the stable binding interfaces between the interacting proteins from the Mago–Y14–EIF4A3–Barentsz–UPF3b (Buchwald et al., 2010), SMG1–SMG8–SMG9–UPF1 (Langer et al., 2020), and eRF3a–eRF1 (des Georges et al., 2014) complexes, along with its selectivity toward mRNA. For the first time, we employed molecular dynamic simulations (MDSs) to investigate full-length SMG1 and eRF1 structures with their respective partners, which play pivotal roles in mRNA surveillance and the degradation of transcripts containing PTCs. Additionally, to evaluate the mRNA-binding specificity of the *eRF1* and *EIF4A3* genes, we conducted *in silico* screening of protein–mRNA complexes. Contributing to this understanding, our study investigates protein–mRNA interaction networks within the NMD machinery, providing a foundation for targeted therapeutic development. By analyzing these complexes, we reveal their structural and dynamic alterations that can contribute to cancer evasion mechanisms. Furthermore, to evaluate how cancer-associated variants affect protein–protein or protein–mRNA networks, we systematically analyzed the mutations in core NMD components (eRF1, EIF4A3, and SMG1; Figure 1). Using cancer-derived mutation data from cBioPortal (Cerami et al., 2012), we assessed their impact on protein affinity change with its respective partners and their influence in internal structural stability. Collectively, our findings reveal how cancer-associated mutations structurally and functionally impair NMD components, uncovering key disease mechanisms. These understandings enable the development of precision therapeutics that are capable of either selectively targeting oncogenic variants or restoring wild-type (WT) protein function.

## 2 Results

To systematically investigate mRNA binding with NMD components, we analyzed the selectivity of nucleotide bases toward key sub-complexes, including the EJC (Mago–Y14–eIF4AIII–Barentsz–UPF3b) (Buchwald et al., 2010), SMG1–SMG8–SMG9–UPF1 (Langer et al., 2020), and eRF3a–eRF1 (des Georges et al., 2014), using MD simulations. In addition, we comprehensively analyzed cancer mutations that modify the RNA-binding affinity in individual RBP from the NMD process, considering the variants retrieved from the cBioPortal database (Cerami et al., 2012). The thresholds for determining a significant change in binding affinity or structural stability upon inserting point mutations were system-specific, and therefore, they were defined individually for each protein–protein or protein–mRNA complexes.

### 2.1 Influence of somatic mutations over protein–mRNA binding specificity

**FIGURE 2:**
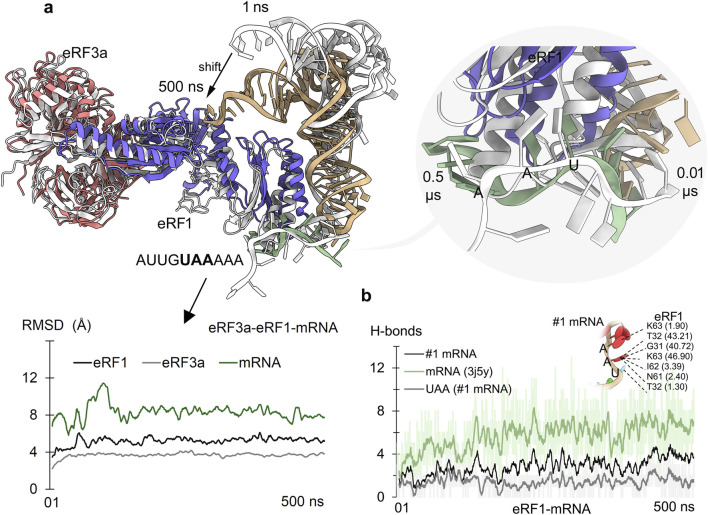
Eukaryotic release factors 1 and 3a (eRF1 and eRF3a) from the NMD machinery and their role in detecting the PTC stop codon. **(a)** Adopted conformations of mRNA upon binding eRF1 during the 500 ns of MD simulation. The right panel represents the mRNA (AUUGUAAAAA; #1 mRNA) containing the UAA stop codon within its fragment. The initial time frame structures are represented in gray, and structures with different colors are from 500 ns. The bottom panel describes the changing stability of protein or mRNA (excluding hydrogen atoms) within the eRF3a–eRF1–mRNA (with stop codon) system. **(b)** Measured eRF1–mRNA hydrogen bond (H-bonds) interactions with mRNA (#1) containing PTC. In addition, direct interactions of UAA nucleotides with eRF1 were measured, along with its residues forming (≥1% occupancy of 500 ns) stable interactions with mRNA. Hydrogen bonds were defined using a donor–acceptor distance cutoff of 3.5 Å and a donor–H–acceptor angle cutoff of ≥160°.

In eukaryotic translation, peptide chain elongation stalls when a stop codon UAA, UAG, or UGA or a PTC reaches the ribosomal A site. There, it is decoded by the class-I release factor eRF1. Following recognition, the class-II release factor (eRF3) triggers hydrolysis of peptidyl transferase RNA from the nascent polypeptide chain at the ribosome. The principal mechanism that eRF1 uses for stop codon recognition has still not been established; however, its ability to recognize all three of the stop codons has been reported. Herein, we evaluated the selectivity and binding of the eRF1–eRF3a complex with the mRNA. Alongside investigating the specificity of eRF1 with all three of the stop codons, we studied the structural effects of cancer-derived protein mutations that can have the potential to modulate the stop codon recognition (Figure 2). In particular, Figure 2a shows the dynamic changes of the eRF3a–eRF1 complex (des Georges et al., 2014) with mRNA (UAA) from the initial and the final time-scale of MD simulation. The stability of individual components within the eRF3a–eRF1–mRNA complex was evaluated using root mean square deviation (RMSD). The MD simulation revealed that the system reached equilibrium by the end of the MD simulation, with both eRF1 and eRF3a proteins exhibiting stability. However, during the initial time frame, the fluctuating RMSD of the mRNA (containing the stop codon) corresponded to a lower number of hydrogen bonds with eRF1 (Figure 2).

**FIGURE 3:**
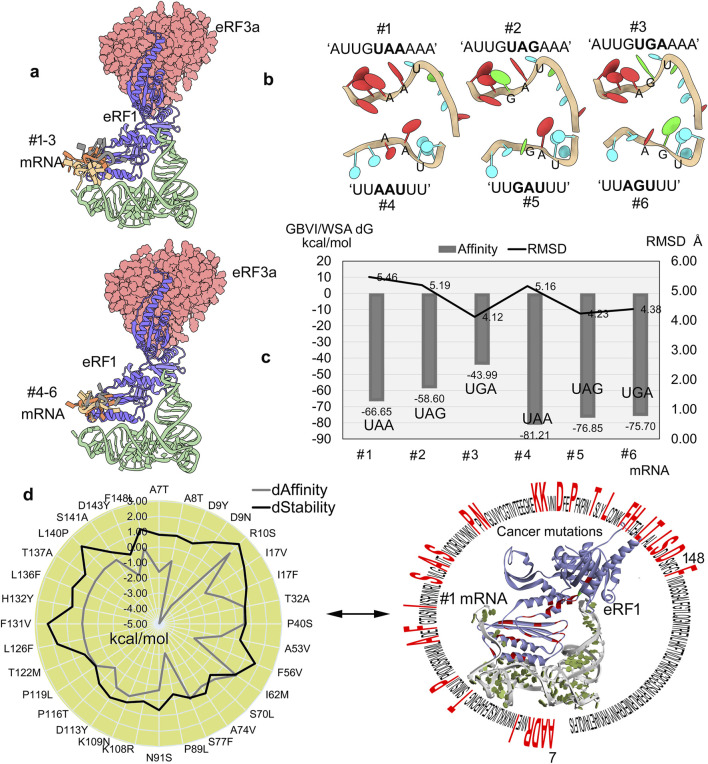
Selectivity and binding specificity of mRNA with eRF1. **(a)** Different mRNA fragments clustered over the eRF1–eRF3a complex. **(b)** Modeled structures of different fragments of mRNA containing the PTCs (UAA, UAG, or UGA) based on the structures extracted from pdb id.: 2xb2 (Buchwald et al., 2010) and 3j5y (des Georges et al., 2014). **(c)** mRNA motifs docket with the eRF1–eRF3a system, and the high affinity motifs were ranked using the GBVI/WSA dG (kcal/mol) scoring function. Furthermore, to assess stability, the RMSDs of individual mRNA motifs were calculated for conformations with high binding affinity. **(d)** Impact of cancer-associated mutations on eRF1–mRNA binding assessed by changes in the affinity and structural stability was evaluated, while the right panel highlights the localization of mutated eRF1 residues within the protein structure. dAffinity and dStability, represent the change in the binding affinity (∆∆G; kcal/mol) with mRNA and the protein folding or stability (∆∆G; kcal/mol), respectively, compared to the wild-type, respectively. Increasingly negative ΔΔG values indicate stabilizing mutations that enhance structural integrity or binding affinity, while positive values correspond to destabilizing effects.

Time-dependent eRF1–mRNA dynamics revealed that mRNA containing PTCs stabilized its binding with eRF1 by the end of MD simulation; however, it was concomitant with different conformational shifts. Notably, the stop codon nucleotides from the mRNA motifs contributed substantially to this affinity modulation (Figures 2b, 3a). In particular, the G31, T32, N61, l62, and K63 residues formed stable interactions with mRNA stop codons, defining the surface hotspots of the *eRF1* gene (Figure 2b). Our observations are supported by extensive mutagenesis data (Frolova et al., 2000; Bertram et al., 2000; Inagaki et al., 2002), reinforcing the crucial role of eRF1’s N-domain structural elements: the NIKS motif (61 aa–64 aa), YxCxxxF motif (125 aa–131 aa), and GTS loop (31 aa–33 aa) in stop codon recognition and decoding (Cheng et al., 2009; Bensaude et al., 2024; Liang et al., 2005).

The stop codon-embedded mRNAs (UUAAUUU, UUGAUUU, and UUAGUUU) consistently displayed a higher eRF1 affinity than AU-rich (AUUGUAAAAA, AUUGUAGAAA, and AUUGUGAAAA) (Figure 3c) motifs. The mRNAs containing PTCs exhibited a stable conformation, and, in particular, these stop codon embedded motifs showed a declining binding affinity (generalized-born volume integral/weighted surface area (GBVI/WSA dG); kcal/mol) trend with eRF1, that is, the highest with UAA and the lowest with UGA (Figure 3a). However, unlike the binding affinity trends, the internal RMSDs demonstrating the stability of individual mRNA motifs exhibited slight fluctuations (Figure 3c). Comparing the RMSDs and binding affinity revealed that AU-rich motifs (#1 to #3 mRNAs) exhibited higher RMSD values (indicating less stable conformations), which may have resulted in slightly weaker affinity with eRF1, than other stop codon-containing transcripts (#4 to #6 mRNA; Figure 3c). The eRF1 is crucial for translation termination via identifying all three stop codons with triggering polypeptide release, and its dysregulation can lead to readthrough events, proteotoxic stress, and oncogenic protein production. For example, the R137C/Q mutation in eRF1 can hinder its binding with mRNA containing PTCs, which results in induced translational readthrough (Nůsková et al., 2021).

Considering the crucial roles of different eRF1 residues interacting with mRNA or PTC recognition that might affect the functional properties of this protein, the effect of cancer-derived mutation over the protein–RNA affinity was investigated. To evaluate such structural properties, the optimized protein–mRNA coordinates after 500 ns MD simulation (Figures 2, 3) were retrieved. In particular, a set of residues from the NIKS region, the YxCxxxF motif, and the GTS loop responsible for stop codon recognition (Muhlrad and Parker, 1994; Liang et al., 2005) were found to show significant effects upon inserting point mutations. The T32A and I62M cancer mutations decreased mRNA binding affinity and destabilized the protein structure. In contrast, the L126F and F131V mutations lack any effect on mRNA binding but reduce the protein’s stability (Figure 3d). In addition, the 7–148 residues from eRF1 were found to extensively interact with mRNA containing PTCs (Figure 3d). Consequently, we evaluated the impact of cancer driver mutations within this region on protein–mRNA binding affinity (Figure 3d). The D9Y and P89L mutations enhanced eRF1–mRNA interaction (∆∆G; with affinity change ≤ −3.00 kcal/mol), whereas R10S and I62M (∆∆G; ≥1.00 kcal/mol) weakened it (#1 mRNA, Figure 3d). Additionally, the R10S, F56V, F131V, and L140P (∆∆G; stability change ≥1.50 kcal/mol) cancer variants destabilized the eRF1 structure (Figure 3d).

Our findings suggest that the activity of eRF1, a key protein in translation termination, can be modulated by cancer-related mutations. A reduction in the binding efficiency between eukaryotic release factor 1 and premature termination codons (PTCs) compromises the translation termination fidelity, leading to translational readthrough (as previously reported (Cheng et al., 2009; Baradaran-Heravi et al., 2021; Gurzeler et al., 2023; Pillay et al., 2016)). When a near-cognate tRNA outcompetes eRF1 in stop codon recognition, the readthrough process is activated, extending the protein synthesis elongation phase (Mangkalaphiban et al., 2021). This allows ribosomes to bypass the stop codon, generating aberrant C-terminally extended proteins that may promote oncogenesis. In this line, the cancer-associated mutations in eRF1 can also disrupt its interactions (e.g., UPF1 and eRF3), further impairing translation termination and potentially contributing to tumorigenesis (Pillay et al., 2016). Conversely, eRF1’s role in nonsense-mediated decay has made it a promising therapeutic target. Small molecules designed to degrade eRF1 (Cheng et al., 2009; Baradaran-Heravi et al., 2021) or inhibit its function (Gurzeler et al., 2023) could restore protein expression by suppressing premature termination at PTCs.

### 2.2 Influence of cancer-derived mutations over the selectivity of EJC toward mRNA binding

The core EIF4A3 protein from the exon–junction complex primarily forms interactions with the spliced mRNA in a sequence-independent but structure-dependent manner. These resulting spliced complexes, processed by the spliceosome, are positioned on the mRNA during pre-mRNA splicing (Buchwald et al., 2010; Wang et al., 2018). It has been reported that some interferon-β intron-less mRNAs can be recruited through alternative mechanisms (Wyce et al., 2007). EIF4A3 plays a crucial role within the NMD process that degrades mRNAs containing PTCs, which, if translated, produce truncated proteins (Meznad et al., 2021). As a first step, the entire NMD process is triggered when a PTC is located >50–55 nucleotides downstream of the last EJC. It has been reported that if the interactions of EIF4A3 is altered, it can impair signaling to NMD and alter the degradation process of mRNAs containing PTC (Gehring et al., 2005). The ATP-dependent RNA helicase activity of EF4A3 engages the recruitment of UPF proteins within NMD, and mutations can fail to recruit such factors, eventually suppressing NMD. Dysregulation of EIF4A3 has a direct correlation with tumor aggressiveness and poorer survival (López-Cánovas et al., 2022). Moreover, it could be proposed that targeting EIF4A3 within the NMD machinery could offer novel therapeutic avenues in cancers that can induce defective RNAs. Although EIF4A3 does not directly interact with stop codons unlike the eRF1 or *eRF3* genes, it plays a critical role in stop codon recognition within the NMD machinery. This spatial protein–mRNA relationship determines the fate of PTC-containing or aberrant mRNAs.

**FIGURE 4:**
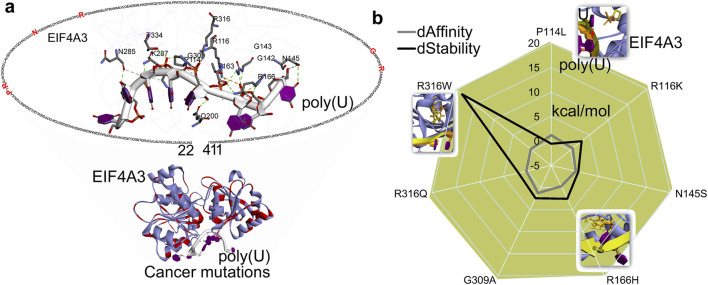
Interaction patterns and docking of mRNA fragments with the EJC complex. **(a)** Network of poly(U) (pdb id.: 2xb2 (Buchwald et al., 2010)) with EIF4A3 and mutated in different cancer types. **(b)** Cancer-derived mutations introduce global structural perturbations in EIF4A3–mRNAs, altering the protein’s binding affinity and stability. In addition, a set of protein–mRNA interactions that significantly impact these interaction patterns are highlighted. dAffinity and dStability represent the change in the affinity (∆∆G binding; kcal/mol) or stability (∆∆G; kcal/mol), respectively, compared to the wild-type. Increasingly negative ΔΔG values indicate stabilizing mutations that enhance structural integrity or binding affinity, while positive values correspond to destabilizing effects.

These crucial roles of EIF4A3 make it an important factor within the PTC recognition and degradation processes, and its dysregulation or overexpression has been implicated in cancer progression or tumorigenesis (Tatsuno et al., 2019; Shah et al., 2020). The analysis of protein–mRNA interactions from the crystal structure (Buchwald et al., 2010) revealed that P114, R116, G142, G143, N145, T163, R166, Q200, N285, K287, G309, R316, and T334 residues directly interact with poly(U) mRNA (Figure 4a). Cancer-derived mutations in these interacting residues were retrieved from cBioPortal (Cerami et al., 2012) and evaluated for their impact on EIF4A3–mRNA binding affinity or structural stability (Figure 4b). Among studied mutations, the P114L and G309A variants significantly hindered the protein–mRNA interactions (∆∆G; affinity change ≥1.00 kcal/mol). In addition, the R116K, R166H, G309A, and R316W mutations significantly destabilized the protein structure (∆∆G; stability change ≥2.00 kcal/mol; Figure 4b). An analysis of the conformational dynamics of individual mRNA nucleotides and the EIF4A3 P114L mutant suggests that such amino acid substitution disrupts the mRNA positioning required for productive interaction (Figure 4b). Evaluation or prediction of cancer mutations that reduce or induce eIF4A3’s affinity for mRNA shall contribute to better molecular-level understanding of these systems.

Moreover, the yeast eukaryotic initiation factor (4A) has been extensively studied using MD simulations under different conditions, and, in particular, the closed conformation (as shown in Figure 4) is found to be more stable than the open state (Meng et al., 2014; Essegian et al., 2021). The superimposition of human EIF4A3 and yeast eIF4A revealed high structural similarity in conserved motifs, sharing 62% sequence similarity (Meng et al., 2014). In WT systems (Meng et al., 2014), cancer-associated mutations (P114L, R116K, R166H, G309A, and R316W) that influence protein–mRNA interaction (Figure 4b) showed a stable RMSF, specifically in the closed conformation of yeast eIF4A (structurally similar to human eIF4A3) upon binding ATP and mRNA. In addition, the free energy calculations (Meng et al., 2014) revealed two pathways (ATP binding first, then mRNA, or *vice versa*); the mutual binding of ATP and mRNA gained hydrogen bonds, triggering the allosteric process. The fact that the cooperative binding of ATP and RNA facilitates this conformational change has been reported previously, supported by hydrogen bond analysis during the allosteric modulation of protein (Meng et al., 2014). Our study assesses the impact of mutations on the X-ray diffraction EIF4A3 structure (Figure 4) in its closed conformation with ATP and mRNA. To evaluate their influence on the allosteric regulation of human EIF4A3, we recommend simulating each mutation individually in the presence or absence of ATP and mRNA.

### 2.3 The dynamic interactions of SMG1 with components of the NMD machinery

**FIGURE 5:**
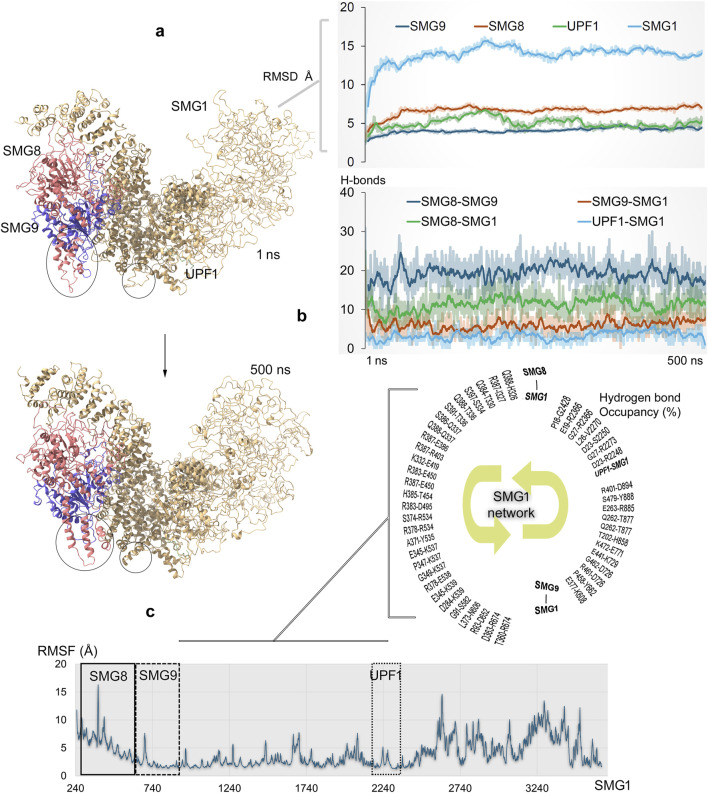
Dynamics of full-length SMG1 model structure and its interactions with different components (SMG8, SMG9, and UPF1) within the NMD process (Padariya et al., 2024). **(a)** Simulated SMG1–SMG8–SMG9–UPF1 or SURF complex over the 500 ns time scale. Significant structural changes in the proteins are marked with a circle. The right panel demonstrates the RMSD of individual components within the SMG1–SMG8–SMG9–UPF1 system. **(b)** Protein–protein interaction (PPI) affinity within the SMG1 complex, which was computed based on the number of hydrogen bonds. The bottom panel represents high occupancy (≥10%) interactions between proteins involved in docking with the SMG1 kinase. Hydrogen bonds were defined using a donor–acceptor distance cutoff of 3.5 Å and a donor–H–acceptor angle cutoff of ≥160°. **(c)** RMSFs (root mean square fluctuations) demonstrate the flexibility or stability of individual SMG1 residues along with highlighted regions having PPIs with *SMG8*, *SMG9*, and *UPF1* genes.

The SMG1 kinase demonstrated itself as a crucial component within the NMD machinery that participates in degrading the mRNA containing PTCs (Kurosaki et al., 2016). SMG8 and SMG9 are the two regulatory proteins directly interacting with SMG1 (Langer et al., 2020; Zhu et al., 2019), and they are involved in the phosphorylation of UPF1 (Figure 1). The mechanisms of SMG1-interacting proteins are essential for preserving mRNA integrity and blocking the synthesis of aberrant, truncated proteins. The global structural response and dynamics of SMG1 in the presence of its interacting partners was evaluated using the MD simulation technique. Moreover, SMG1 dysregulation has been implicated in multiple cancer types, where it directly impacts NMD-mediated quality control, DNA repair mechanisms, and cell survival pathways (Padariya et al., 2024; Roberts et al., 2013). Key protein–protein interaction (PPI) residues within the SMG1 network, which are frequently mutated in various cancers, were computationally analyzed (Figure 5).

During MD simulation, the RMSDs of individual components from the SMG1–SMG8–SMG9–UPF1 system reached stable values by the end of simulation, indicating overall protein stability. However, SMG1 exhibits higher RMSD values, likely due to its large size, and these fluctuations primarily occur in the region spanning residues ∼2,500–3,661. The simulations suggest that the absence of stabilizing binding partners (SMG8, SMG9, or UPF1) in this region may have contributed to the observed high fluctuations over the 500 ns MD trajectory. The dynamics of full-length SMG1 with SMG8 and SMG9 proteins revealed the positioning of key residues and identified protein surfaces that contribute in forming hotspot profiling based on their PPIs. Several conformations within the SMG1 network were observed during the MD simulation, and the visible shifting in F2215–S2250 residues was observed in particular (Figure 5a). While SMG1 undergoes structural folding from its center to the C-terminal region, the *SMG9* gene exhibits conformational changes around the D490–W559 residues (Figure 5a). From our findings, it could be proposed that such dynamic changes within the SMG1 structure may regulate its activation and inactivation when complexed with the *SMG8*, *SMG9*, and *UPF1* genes (Zhu et al., 2019). Constructed PPI sites revealed that SMG8–SMG9 has a higher number of networks, and SMG1 forms major interactions with SMG8 compared to those with SMG9 (Figure 5B). Individual SMG1 residues making high-occupancy (≥10% of 500 ns MDS) interactions with its partner proteins were identified, which contributed in designing hotspot regions (or interface residue mapping) over these protein components (Figure 5b). A sequential interaction pattern was observed between SMG1 and its partners; that is, residues 326–674, 608–894, and 2248–2428 were involved in binding with SMG8, SMG9, and UPF1, respectively. These SMG1-interacting regions exhibited stable conformations (low RMSF; root mean square fluctuations) throughout the MD simulation (Figure 5c).

**FIGURE 6:**
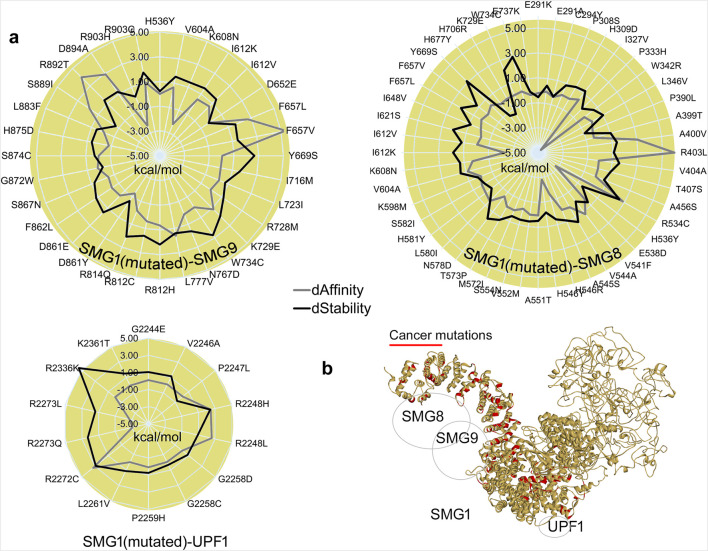
Structural influence of cancer variants over the SMG1 model, and its changing interactions with *SMG8*, *SMG9*, or *UPF1* upon inserting point mutations. **(a)** Change in the binding affinity between protein–protein interactions, and the SMG1 structure stability change upon the insertion of mutations. **(b)** Cancer variants represented over the SMG1 structure that we found showing a significant effect in binding with its partner proteins. dAffinity and dStability represent the change in the affinity (∆∆G binding; kcal/mol) or stability (∆∆G; kcal/mol), respectively, compared to the wild-type. Increasingly negative ΔΔG values indicate stabilizing mutations that enhance structural integrity or binding affinity, while positive values correspond to destabilizing effects.

The SMG1 kinase attained a stable conformation with its binding partners by the end of MD simulation. Given that SMG1 is frequently mutated across various cancers (cBioPortal (Cerami et al., 2012)), we extracted the protein coordinates at 500 ns to evaluate the effects of point mutations on the SMG1–SMG8, SMG1–SMG9, and SMG1–UPF1 complexes (Figure 6). In particular, the cancer-derived mutations within SMG1 located at 248–910 and 2200–2400 amino acids were evaluated in terms of the change in affinity or stability in the presence of SMG8/SMG9 and UPF1, respectively. Variants within the SMG1 region (291 aa–737 aa) significantly affected the binding affinity with SMG8. Notably, A400V, R403L, and R534C mutations impaired SMG1–SMG8 binding (∆∆G; affinity change ≥2.50 kcal/mol), whereas P333H, H536Y, H546Y, and I612K (≤2.30 kcal/mol) enhanced protein–protein affinity (Figure 6). In addition, the R534C, Y669S, and F737K (∆∆G; stability change ≥2.50 kcal/mol) mutations destabilized the SMG1 structure (Figure 6). In the SMG1–SMG9 network, the cancer-derived variants in SMG1 ranging 536 aa-903 aa led to significant changes in protein–protein binding affinity.

Specifically, the mutations F657L/V, R892T, and D894A (∆∆G; affinity change ≥2.50 kcal/mol) reduced SMG1–SMG9 binding affinity, whereas K608N, D861Y, and R903H had opposite effects (∆∆G; ≤ −1.30 kcal/mol). SMG1 mutated in the presence of UPF1 revealed that the R2248H/L and R2272C mutants (∆∆G; affinity change ≥2.50 kcal/mol) hindered the PPIs and the variant R2273Q/L (≤2.50 kcal/mol) induced the interactions (Figure 6). For the SMG1–UPF1 complex, residues reducing the protein–protein affinity were found to hinder the stability of the SMG1 structure (Figure 6). The SMG1 kinase within the NMD machinery has a crucial role, and any alteration (cancer mutations) in it disrupts UPF1 phosphorylation, eventually resulting in improper functioning of NMD to detect mRNAs containing PTC. UPF1 phosphorylation by SMG1 acts as a platform to engage endonucleases for the aberrant mRNA degradation process. In addition, it has found interactions with eRF1 and eRF3 translation termination factors for the SURF complex. Inactive SMG1 can lead to stabilization of aberrant mRNAs, resulting in the accumulation of truncated proteins that promote cancer progression (Kashima et al., 2006; Dana and Tuller, 2014; Bongiorno et al., 2021). Moreover, it has been reported that potentially targeting SMG1 can inhibit the NMD process that can result in triggering the immune responses in cancer through enhanced neoantigen presentation (Cook et al., 2025). These predicted effects of SMG1 mutations (induced or reduced affinity) on our studied complex align with their prevalence in cancer genomes, proposing a structural basis for NMD dysregulation. Collectively, our findings demonstrated a prioritized variant (Figure 6) that can merit the investigation of SMG1 as potential biomarkers or drug targets.

## 3 Conclusion

The NMD process relies on RNA-binding proteins, including eRF1, EIF4A3, and UPF1, to either degrade PTC-containing mRNAs or execute quality-control checkpoints. Although PTCs underlie numerous genetic disorders and cancers, no targeted therapies currently exist to modulate this process therapeutically. Despite several efforts applied to evaluate the NMD machinery, details about molecular mechanisms and structural arrangements between different subprocesses of NMD workflow are still lacking. One of the working models highlights that upon recognition of PTCs in the upstream of EJC, the translation is paused, resulting in eRFs recruiting the UPF1 RNA helicase. Herein, implementing MD simulation techniques, we elaborated the structural properties of EJC, eRFs, and SURF components, along with their participation in the recognition and binding to mRNAs containing PTCs. Although our current study demonstrates that the protein–protein or mRNA systems reached stable states, we acknowledge that the absence of replicated MD simulations can be considered when assessing the robustness of our defined structural properties. The mutational effect on the stability and binding of protein–protein or mRNA were characterized by providing novel insights into the molecular mechanisms of the NMD process. Our current approach treats only the mutation site as flexible; this simplification precludes the observation of allosteric perturbations over the protein structure. Systematic MD simulations would be required to map the full structural consequences of these variants.

Structural dynamics of the eRF1–mRNA system revealed that mRNA containing a PTC progressively increased its affinity for eRF1 over time, driven by conformational changes. Notably, the stop codon nucleotides were identified as a major contributor to this affinity shift. From the eRF1–eRF3a–mRNA (UAA) system, the eRF1 residues G31, T32, N61, l62, and K63 were found to stably interact with mRNAs. eRF1 exhibits stronger binding affinity to stop codons embedded within the UUAAUUU, UUGAUUU, and UUAGUUU mRNA fragments than to AU-rich motifs (AUUGUAAAAA, AUUGUAGAAA, and AUUGUGAAAA). The UAA, UAG, and UGA stop codons showed a stepwise reduction in eRF1 binding affinity, with UAA having the highest affinity and UGA having the lowest. In particular, the AU-rich motifs that exhibited higher RMSDs resulted in slightly weaker affinity with eRF1 than with other stop codon-containing motifs. Analyzing the effect of cancer-derived mutation demonstrated that residues D9Y, F56V, P89L/R10S, and I62M showed induced/reduced interaction of eRF1–mRNA (UAA). Such impaired eRF1–mRNA binding due to mutations may enable the stop codon readthrough by the ribosomes, yielding oncogenic C-terminal protein extensions. Cancer-linked eRF1 variants may also destabilize interactions with binding proteins from the NMD process, exacerbating the termination defects and tumorigenic potential. Similarly, due to the mRNA-binding potency of EIF4A3 (a core component of the EJC), it is recognized as a critical factor in PTC recognition and mRNA degradation. Although EIF4A3 lacks direct contact with the PTC, its ATP-dependent RNA helicase activity facilitates the recruitment of UPF proteins during NMD. Alterations within the EIF4A3 gene can impair this recruitment, ultimately suppressing NMD, and it has been linked to cancer progression. Notably, our *in silico* predictions demonstrated that different cancer-derived variants such as P114L and G309A disrupt protein–mRNA interactions, whereas R116K, R166H, G309A, and R316W destabilize the protein structure. These mutations were found to induce conformational changes within the mRNA nucleotides, thus obstructing the binding sites required for proper protein recruitment.

Investigating the dynamics of SMG1–SMG8–SMG9 with UPF1 helicase (potentially binding mRNA), the interaction hotspot profiling between these components was constructed. For the residue range F2215–S2250 in SMG1, a conformational shift was recorded alongside its structural packing from the center to the C-terminal region. Such flexibility or the dynamic changes of SMG1 can reflect its activation and inactivation when complexed with SMG8, SMG9, and UPF1. Constructed PPI sites revealed that SMG1 forms major interactions with SMG8 compared to those with SMG9. In addition, the effect of cancer-derived mutations in SMG1 (located within 248 aa–910 aa and 2,200 aa–2,400 aa) was characterized, and their effects on the binding affinity and stability in the presence of SMG8, SMG9, and UPF1 were predicted. Notably, mutations such as A400V, R403L, R534C, F657L/V, R892T, D894A, R2248H/L, and R2271C significantly impaired SMG1’s interaction with SMG8, SMG9, or UPF1. Such disruptions by cancer mutations can potentially inhibit UPF1 phosphorylation by the SMG1 kinase, compromising the NMD machinery’s ability to detect PTC-containing mRNAs and eventually leading to cancer progression. We believe that our findings shall provide a valuable foundation for experimental validation, along with the targeted selection of specific mutations. To enhance translational relevance, experimental approaches such as the reporter assays (for stop codon readthrough), RNA immunoprecipitation (to assess mRNA binding), or co-immunoprecipitation (to evaluate SMG1 complex assembly) could be employed. Overall, in this study, we emphasize the therapeutic modulation of NMD components, which represents a promising multi-target approach for cancers with defective mRNA metabolism. EIF4A3 targeting could restore RNA surveillance, and eRF1 inhibition may enable PTC readthrough to recover functional proteins, whereas SMG1 suppression could enhance immunogenicity through neoantigen presentation. Such a combinatorial strategy can simultaneously address both genetic restoration and immune activation, offering synergistic potential for precision oncology.

## 4 Materials and methods

Individual full-length structures of human SMG1, SMG8, and SMG9 (Langer et al., 2020) were constructed using the SWISS-MODEL tool (Waterhouse et al., 2018), which implements homology modeling techniques for inserting missing residues. Furthermore, these models were superimposed to the SMG1–SMG8–SMG9 crystal structure bound to UPF1 (pdb id.: 6z3r) (Langer et al., 2020) using the BIOVIA Discovery Studio (Dassault Systèmes, BIOVIA Corp., San Diego, CA, United States) pipeline, while maintaining the positions of individual components within the complex. Superimposing each modeled component suggests a good correlation with available crystal structures (Langer et al., 2020). For the SMG1–SMG8–SMG9–UPF1, EJC (Buchwald et al., 2010), and eRFs (des Georges et al., 2014) systems, energy minimization was performed with the CHARMM27 forcefield (Bjelkmar et al., 2010) in the Molecular Operating Environment (MOE; Chemical Computing Group Inc., Montreal, QC, Canada) package. To detect the active site regions in the generated structures, an alpha sphere-based approach was used, which generates pseudo-atoms or alpha-spheres based on Voronoi tessellation of the protein surface and accurately predicts the docking spaces (Schmidtke et al., 2010; Kudo et al., 2023). Individual protein and mRNA structures were analyzed in ChimeraX (Pettersen et al., 2021), BIOVIA Discovery Studio (Dassault Systèmes, BIOVIA Corp., San Diego, CA, United States), and MOE (Chemical Computing Group Inc., Montreal, QC, Canada) packages.

### 4.1 The simulation protocols for protein–protein or protein–mRNA complexes

The modeled protein structures for the eRF3a–eRf1, EJC, and SMG1–SMG8–SMG9–UPF1 components were subjected to primary structure filtering, which was performed using a short MD simulation applying the CHARMM27 forcefield (Bjelkmar et al., 2010) within the MOE (Chemical Computing Group Inc., Montreal, QC, Canada) package. The resulting eRF3a–eRf1 (mRNA containing PTC) and SMG1–SMG8–SMG9–UPF1 protein or mRNA coordinates were used for further extensive MD simulations using the GROMACS 4.6.5 software (MacKerell et al., 1998) and the CHARMM27 forcefield (Bjelkmar et al., 2010). Although recent advances in forcefields have demonstrated significant progress, we opted to use CHARMM27 throughout our study to maintain consistency and ensure compatibility across all analyses, including MD simulations, protein–mRNA docking, and mutation landscape assessments. For MDS, the simple point charge SPC water condition (Berendsen et al., 1981) was selected during the topology process, and the appropriate number of Na+ and Cl- ions was added to the system for charge equilibration. The protein was placed in a 10 Å-thick dodecahedron simulation box, and the periodic boundary conditions (PBCs) were applied to generate the crystal environment. The systems were energy minimized using the steepest descent algorithm, and the long-range electrostatic interactions were treated using the Particle Mesh Ewald (PME) algorithm (Darden et al., 1993). All covalent bonds were constrained using the LINCS algorithms (Darden et al., 1993). After the system equilibration for 1,000 ps, the NPT (isobaric–isothermal ensemble simulation) production runs were performed. The temperature and pressure coupling were enforced with the V-rescale thermostat (Bussi et al., 2007) and the Parrinello–Rahman barostat (Parrinello and Rahman, 1981) using 300 K temperature and 1 bar pressure, respectively. In total, the MD simulations were carried out for 500 ns using the leapfrog integrator (Van Gunsteren and Berendsen, 1988), and the atom coordinates were saved every 10 ps. All systems were analyzed using the GROMACS package and visual molecular dynamics (VMD) tools (Humphrey et al., 1996). As a H-bonding criterion, a donor–acceptor atom cutoff distance of 3.5 Å and an intermolecular donor–H–acceptor H-bonding angle cutoff ≥160°–180° was considered.

### 4.2 Screening of mRNA motifs and mutational landscape within the NMD components

The crystal structure of eRF3a–eRF1–mRNA (pdb id.: 3j5y) (des Georges et al., 2014) was retrieved from the pdb database (www.rcsb.org) (Rose et al., 2011). The EJC complex (Mago–Y14–eIF4AIII–Barentsz–UPF3b; pdb id.: 2xb2) (Buchwald et al., 2010) with poly(U) was energy minimized by using the CHARMM27 forcefield and the default parameters within the MOE package (Chemical Computing Group Inc., Montreal, QC, Canada). For the eRF3a–eRF1–mRNA (pdb id.: 3j5y (des Georges et al., 2014)) system, molecular docking was performed using the MOE pipelines, and the coordinates retrieved from the optimized (MD simulated) structures were docked with six different mRNA motifs consisting of PTCs, namely, AUUGUAAAAA (#1), AUUGUAGAAA (#2), AUUGUGAAAA (#3), UUAAUUU (#4), UUGAUUU (#5), and UUAGUUU (#6). The protein active sites were predicted using the “Alpha Shapes” construction geometric method of MOE modules, which computes the possible recognition sites of the protein and classifies them as either “hydrophobic” or “hydrophilic” (Edelsbrunner, 1995). Comparative structural analysis reveals that the predicted active sites in our eRF1 model align with our MD simulation-identified mRNA binding regions in the eRF3a–eRF1 complex and the experimentally determined binding interfaces from cryo-EM (pdb id.: 3j5y (des Georges et al., 2014)). Individual protein–mRNA binding affinities were computed using the force field-based GBVI/WSA dG (CHARMM27) scoring function, which demonstrates an implicit solvent model. The receptor–mRNA docking was performed using the “triangle matcher” algorithm, maintaining receptor rigidity while allowing mRNA flexibility, generating 1,000 poses or conformational per individual mRNAs. Docking parameters included convergence gradient tolerance of 0.01, a maximum of 500 iterations per run, pharmacophore constraints with 100 force constant, and 0.4 Å radius offset. All motifs docked with the eRF3a–eRF1 complex were ranked using the GBVI/WSA dG (kcal/mol) scoring function to identify mRNAs with the best affinity to eRF1.

Cancer-derived mutations (retrieved from the cBioPortal database (Cerami et al., 2012)) in all three complexes, SMG1–SMG8–SMG9-UPF1 (pdb id.: 6z3r (Langer et al., 2020)), Mago–Y14–eIF4AIII–Barentsz–UPF3b (pdb id.: 2xb2 (Buchwald et al., 2010)), and eRF1–eRF3a; (pdb id.: 3j5y (des Georges et al., 2014)), were subjected to the “residue scan” protocol of the protein design module in the MOE package. The “residue scan” protocol in MOE computes changes in the locally minimized conformational ensemble upon mutation by generating random amino acid substitutions, followed by side-chain repacking of neighboring residues. For each single mutation, the structural model with a thorough conformational ensemble was generated using the “LowMode MD” ensemble and CHARMM27 forcefield with default parameters. The rotamer explorer was configured with an RMSD cutoff of 0.25 Å, the energy window was set to 10 kcal/mol, and the residues within the 4.5 Å distance of a residue to be mutated were treated as flexible and the other residues were kept rigid or fixed. In particular, 10,000 search conformations were carried out, and the ∆stability (kcal/mol) was calculated as the relative binding free energy difference (∆∆G bind; kcal/mol) between the mutant (∆G) and WT (∆G). The sign and magnitude of ∆∆G directly reflect the mutational effects: an increasingly negative ΔΔG value signifies greater stabilization or active mutations (<0; ∆∆G stability or affinity cutoff and ΔG mutant > ΔG wild), and a positive ΔΔG value reveals progressively stronger destabilization of the protein structure (ΔG mutant < ΔG wild) (Eriksen et al., 2014).

The structural perturbations caused by each mutation or cancer variant (mutation expression) (Kitchen et al., 2004) were computed with the aim of obtaining the stability (∆stability or dstability, or ∆∆G; kcal/mol) or binding free energy (∆∆G bind or daffinity; kcal/mol) trade-offs when the mRNA motif binds to the WT and mutated protein complex. The ∆∆G value represents the mutation-induced change in thermostability relative to the WT protein, which was calculated by statistically averaging the free energy differences across all sampled conformations using Boltzmann weighting factors (Eriksen et al., 2014). While protein engineering offers powerful opportunities to tailor protein properties, the exponential complexity of sequence space necessitates intelligent search strategies. Current approaches combine biophysical principles with evolutionary data to focus mutagenesis sites on the most promising regions of sequence space. However, such high-throughput techniques typically compute mutational effects only at the active sites of a protein structure, thereby failing to capture potential global allosteric perturbations of a gene. To comprehensively identify allosteric effects resulting from mutations, MD simulations are recommended as they can probe long-range conformational changes across the entire protein structure.

## Data Availability

The original contributions presented in the study are included in the article/Supplementary Material; further inquiries can be directed to the corresponding author.
